# Recurrent opportunistic infections in a HIV-negative patient with combined C6 and *NFKB1* mutations: A case report, pedigree analysis, and literature review

**DOI:** 10.1515/med-2024-1019

**Published:** 2024-12-31

**Authors:** Yamei Zheng, Liwen Guan, Jiao Li, Yihui Fu

**Affiliations:** Department of Pulmonary and Critical Care Medicine, Hainan General Hospital (Hainan Affiliated Hospital of Hainan Medical University), Haikou, Hainan, China; Department of Gastroenterology, Sanya Central Hospital (Hainan Third People’s Hospital), Sanya, Hainan, China; Department of Pathology, Hainan General Hospital (Hainan Affiliated Hospital of Hainan Medical University), Haikou, Hainan, China

**Keywords:** opportunistic infections, C6 mutation, *NFKB1* mutation, primary immunodeficiency disorder

## Abstract

**Introduction:**

Recurrent opportunistic infections are particularly common in patients infected with human immunodeficiency virus (HIV). However, these opportunistic infections have also been reported in HIV-negative patients, especially those with primary immunodeficiency disorder (PID), a condition that involves a large heterogeneous group of disorders arising from defects in immune system development and/or function.

**Case:**

Here, we report a very rare case of recurrent opportunistic infections in a non-HIV-infected patient combined with mutations in complement component C6 and nuclear factor kB subunit 1 (*NFKB1*). The patient first developed *Pneumocystis jirovecii* pneumonia, followed by cytomegalovirus esophagitis. Reduced CD4+ T and B lymphocyte counts, hypogammaglobulinemia were observed. The patient was HIV negative, and congenital immunodeficiency-related genes indicated combined C6 and *NFKB1* mutations. Gene detection was undertaken with blood samples from the patient’s parents and younger brother. None of the family members possessed both gene mutations, suggesting that the simultaneous mutations of C6 and *NFKB1* caused primary immunodeficiency in the patient and resulted in recurrent opportunistic infections. In addition, we performed a review of the relevant literature to assess the clinical manifestations of C6 and *NFKB1* mutations.

**Conclusion:**

A diagnosis of PID should be suspected in patients with recurrent opportunistic infections, decreased CD4+ T and B lymphocyte, and hypoimmunoglobulinemia when secondary immunodeficiency factors can be excluded. In addition, genetic testing of family members should be performed, which may lead to the discovery of novel familial gene mutations.

## 1 Introduction

In clinical practice, acquired immunodeficiency syndrome (AIDS) is often considered in patients suffering with recurrent opportunistic infections, and primary immunodeficiency disorder (PID) is easily overlooked. PID refers to a large heterogeneous group of disorders that result from defects in immune system development and/or function. PIDs are broadly classified as disorders of adaptive immunity (i.e., T cell, B-cell, or combined immunodeficiencies) or of innate immunity (e.g., phagocyte and complement disorders) [1]. Despite it is important to note that PIDs are distinct from secondary immunodeficiencies that may result from other causes, such as viral or bacterial infections, malnutrition, immunoglobulin (Ig) loss, malignancy, or treatment with drugs that induce immunosuppression [2–4]. Differences can be expressed in T lymphocyte subpopulations and immunoglobulin level. However, the clinical manifestations of PID are similar to those of AIDS, with complex clinical manifestations mainly including opportunistic infections, tumors, and autoimmune diseases. Herein, we report a male patient with recurrent opportunistic infections, reduced CD4+ T and B lymphocyte counts, and hypoimmunoglobulinemia, which ultimately proved PID by genetic testing.

## 2 Case presentation

A 23-year-old young man was admitted to our department on the 1st of April 2021 for recurrent cough and expectoration for 1 month. The patient was a seafarer and presented with a 1-month history of productive cough, accompanied by fatigue, slight shortness of breath but denied chest pain and fever. His past medical history included recurrent tonsillitis. And he had no previous family history of cancer or unsafe sexual behavior.

At the laboratorial examination (Table 1), the initial blood routine count was as follows: white blood cell count, 3,290/µL; neutrophils count, 1,920/µL; lymphocyte count, 630/µL. T lymphocyte subpopulations showed that the ratio of CD3+ T lymphocytes/total lymphocytes and B lymphocytes/total lymphocytes was 61.9 and 2.3%, respectively. The proportion of CD3+ CD4+ T and CD3+ CD8+ T in total lymphocytes was 5.6 and 38.9%, respectively. Blood routine count indicated a significant decrease in lymphocytes and T lymphocyte subsets suggested a drastic reduction in CD4+ lymphocytes, with an absolute value of approximately 35/µL. Furthermore, we detected a marked decrease in the number of B lymphocytes. The serum immunoglobulin levels evaluated by turbidimetric inhibition immunoassay were 15.65 g/L for IgG, 2.0 g/L for IgM, and 3.3 g/L for IgA. His serum immunoglobulin levels were normal at this hospitalization. The patient’s lactate dehydrogenase and fungal beta-glucan (G test) concentrations were 990.1 U/L and 232.7 pg/mL, respectively. His human immunodeficiency virus (HIV) antibody test was negative.

**Table 1:** Laboratory parameters

Laboratory parameters	Value	Value	Reference value
	(Hainan General Hospital)	(Sanya Central Hospital)	
White blood cell	3,290/μL	9,880/μL	3,500–9,500/μL
Neutrophils	1,920/μL	7,300/μL	1,800–6,300/μL
Lymphocyte	630/μL	290/μL	1,100–3,200/μL
CD3+ T cell/total lymphocyte	61.90%	56.80%	50–84%
CD3+ CD4+ T/total lymphocyte	5.60%	2.70%	27–51%
CD3+ CD8+ T/total lymphocyte	38.90%	32.10%	15–44%
B cells/total lymphocyte	2.30%	0.10%	5–18%
CD4+ lymphocytes	35/μL	7.83/μL	—
IgG	15.65 g/L	5.12 g/L	7.5–15.6 g/L
IgA	3.3 g/L	1.15 g/L	0.8–4.5 g/L
IgM	2.0 g/L	0.24 g/L	0.46–3.0 g/L
Lactate dehydrogenase	990.1 U/L	—	120–250 U/L
Fungal beta-glucan	232.7 pg/mL	—	0–100 pg/mL

**Figure 1:**
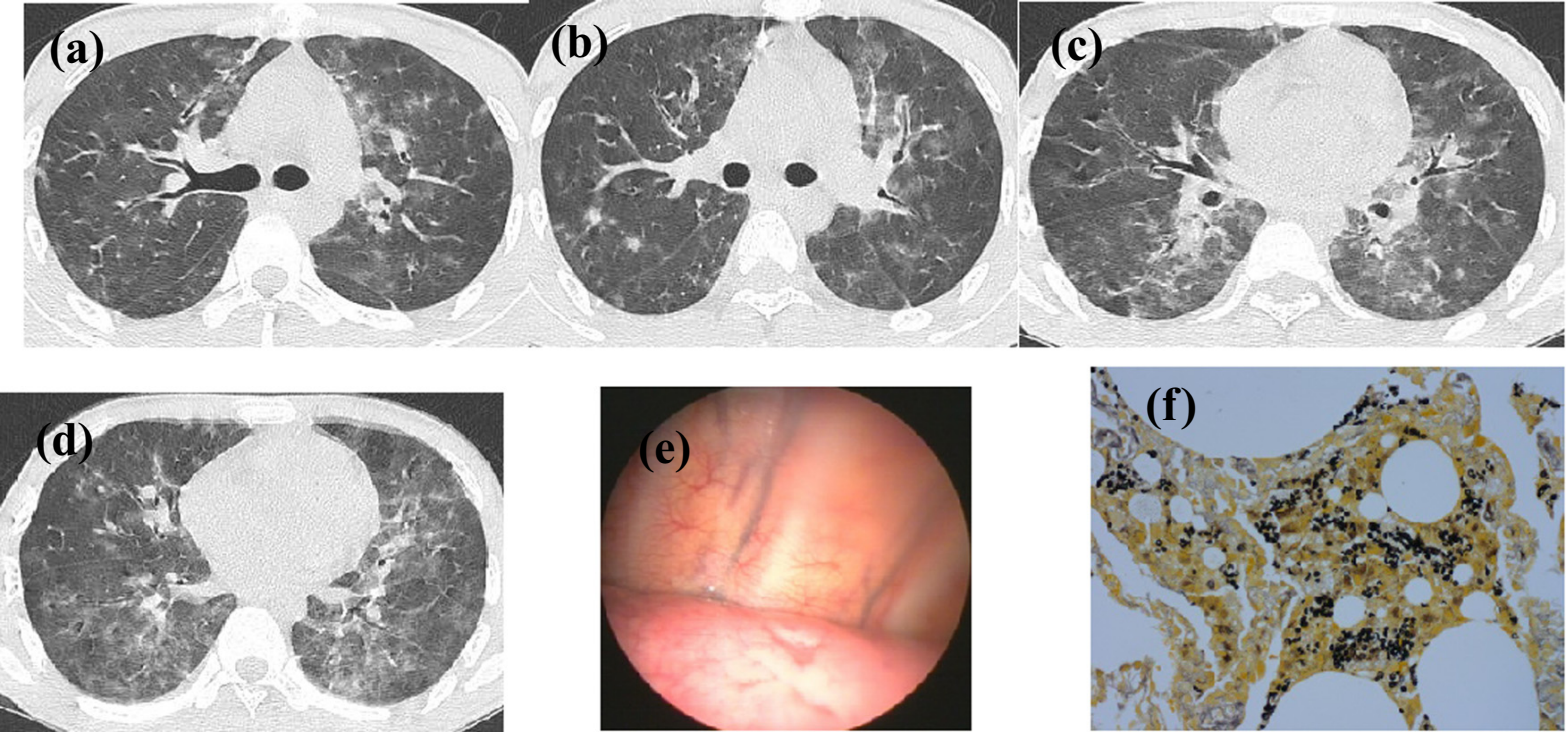
(a)–(d) CT manifestations showing scattered patches and exudates in both lungs. (e) Thoracoscopic findings showing congestion of the parietal pleura, with a smooth surface and focal depression. (f) Grocott stain positivity, with a deflated ping-pong ball-like cyst.

Chest computed tomography (CT) revealed multiple scattered exudative or infectious lesions in both lungs (Figure 1a–d). Pulmonary infection was taken into consideration. Anti-infective treatment with cefmetazole combined with moxifloxacin showed unsatisfactory results. Due to the unclear diagnosis, lung biopsy was performed by medical thoracoscope after artificial pneumothorax. Congestion of the visceral and parietal pleura was observed, with smooth surfaces, focal depression, and no nodules (Figure 1e). Two biopsy samples obtained from the right lower lung lobe and three biopsy samples from the right middle lung lobe were sent for histopathological examination. Pathological analyses of the specimens by medical thoracoscope revealed that the lesions in the right lower and middle lung lobes were consistent with manifestations of *Pneumocystis jirovecii* pneumonia (PJP) (Figure 1f). The patient had experienced respiratory failure during the course of the disease, therefore we prescribed a combination of sulfamethoxazole and caspofungin for anti-infection therapy, while prednisone was administered to reduce inflammation. The patient’s symptoms were relieved and on a follow-up visit 1 month later, a thoracic CT scan demonstrated obvious absorption of the pulmonary inflammation.

Three months later, the patient repeatedly experienced dysphagia, chest pain, and bloody stool, accompanied by dizziness, fatigue, and weight loss; ultimately, he was admitted to Sanya Central Hospital. Blood routine count showed a white blood cell count of 9,880/µL, a neutrophil count of 7,300/µL, and a lymphocyte count of 290/µL.

**Figure 2:**
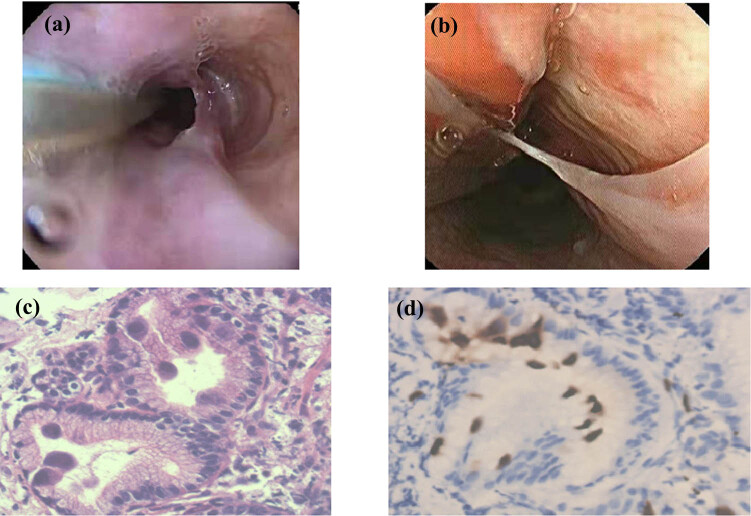
(a) Multiple irregular ulcers with white bulging edges on the esophagus starting from 20 cm away from the incisors, which merge with each other. (b) Close-up view of a deep ulceration. (c) CMV-infected cells in an inflammatory background and in the glands. (d) IHC CMV (+).

**Figure 3:**
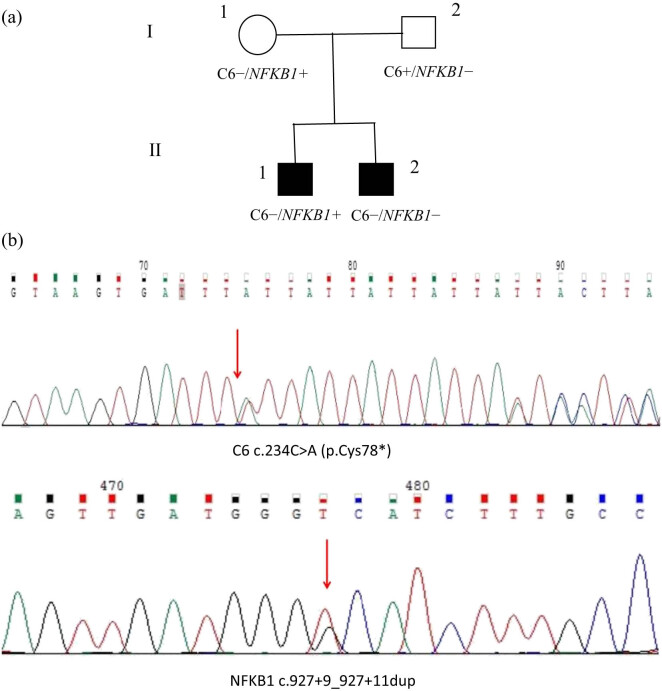
(a) Pedigree of the family (+: wild type; −: heterozygous mutated), Ⅰ-1: mother, Ⅰ-2: father, Ⅱ-1: younger brother, Ⅱ-2: patient. (b) PID-related genes of the patient showed simultaneous gene mutations of C6 and *NFKB1* in the patient.


T lymphocyte subpopulations analysis showed that the ratio of CD3+ T lymphocytes/total lymphocytes and B lymphocytes/total lymphocytes was 56.8 and 0.1%, respectively. The proportion of CD3+ CD4+ T and CD3+ CD8+ T in total lymphocytes was 2.7 and 32.1%, respectively. The absolute value of CD4+ lymphocytes was approximately 7.83/µL. The serum immunoglobulin levels were 5.12 g/L for IgG, 0.24 g/L for IgM, and 1.15 g/L for IgA. The laboratory tests for this hospitalization are illustrated in Table 1. His serum immunoglobulin levels were obviously reduced compared to the results from the first hospitalization. Significant decreases were observed in CD4+ T lymphocyte count and B lymphocyte count. Gastroscopy revealed multiple irregular ulcers with white bulging edges on the esophagus, starting from 20 cm away from the incisors, which merged with each other (Figure 2a). Deep ulcers were identified upon close observation (Figure 2b). Histopathological manifestation of the esophagus by gastroscopic biopsy indicated mucous glands in some areas. Additionally, we identified tissue necrosis, the proliferation of fibrous connective tissue and small blood vessels, and the infiltration of many acute and chronic inflammatory cells in some areas, among which, we observed scattered cells with enlarged nuclei (Figure 2c). The immunohistochemical results were as follows: cytomegalovirus (CMV) (+) (Figure 2d), CD68 (+), CK (−), CK8/18 (−). Pathological diagnosis of the esophagus was consistent with CMV infection. Esophageal tissue was obtained to perform metagenomic next-generation sequencing for the detection of genes of the pathogenic microorganisms. A total of seven human CMV (dsDNA) sequences were obtained, with a high level of confidence. A diagnosis of CMV esophagitis was made. Colonoscopy was not performed owing to the poor general condition of the patient. Due to recurrent opportunistic infections (PJP and gastrointestinal CMV infection) and repeated negativity for HIV antibodies, we considered the differential diagnoses of immunocompromised or immunodeficient conditions associated with opportunistic infections. We did not exclude PID. Comprehensive investigations were necessary to verify primary immunodeficiencies. Further inquiries relating to the patient’s medical history revealed consanguineous marriage of the parents within three generations. Furthermore, his own parents were siblings. Sequences analysis of PID-related genes obtained from the patient, his parents, and his younger brother revealed simultaneous gene mutations of C6 and *NFKB1* in the patient (Figure 3a) but no simultaneous gene mutations in his family members (Figure 3b). The patient experienced CMV esophagitis. Antiviral treatment with ganciclovir was provided, as well as acid suppression therapy, hemostasis, and nutritional support. However, the patient’s symptoms did not improve and he developed a perianal abscess, repeated bloody stool and gastrointestinal hemorrhage. Repeated bloody stool persisted after treatments including anti-infection therapy, drug administration, hemostasis with interventional embolization, and blood transfusion. The patient eventually died.

**Informed consent:** Written informed consent was obtained from the patient’s parents to publish this article.

## 3 Discussion

Clinically, PJP and CMV esophagitis occur mainly in the immunocompromised population and secondary immunodeficiency, including AIDS, a serious disease caused by HIV infection. In HIV patients, when the absolute count of CD4+ T lymphocytes is below 200/μL, PJP should not be ignored and CMV infection must be taken into consideration under the condition that the absolute count of CD4+ T lymphocyte is below 100/μL [5]. Opportunistic infections occurred in patients with severe CD4 depletion [6]. The relationship between immunodeficiencies and “infection” has increasingly been recognized. PID refers to a group of rare disorders that occur mainly due to single gene variations. In such cases, gene mutations lead to functional defects in immune cells and molecules, resulting in insufficiency or absence of immune functions, which manifest as increased susceptibility to infectious, autoimmune, autoinflammatory, and allergic diseases and/or malignant tumors. Immunodeficiency is classified as antibody deficiency, cellular immunodeficiency, and complement deficiency, in addition to deficiencies in the number or function of innate phagocytes [7]. Most PIDs arise from inherited defects in immune system development and/or function; however, acquired forms have also been described, such as neutralizing anti-interferon-γ autoantibody-associated immunodeficiency (which has been noted in over 95% of patients with disseminated infections by nontuberculous mycobacteria) [8]. Many types of PID have been identified and the manifestation of T lymphocyte subsets and the immunoglobulin levels also differs from that of AIDS.

The *NFKB1* gene encodes nuclear factor of κ light polypeptide gene enhancer in B cells 1 (NF-κB1), one of the five REL homology domain-containing proteins. The nuclear factor-kB (NF-kB) signaling pathway plays a major role in mediating multiple cellular events including immune and inflammatory responses, lymphocyte development, cell growth, and programmed death [9]. Recently, heterozygous mutations in *NFKB1* resulting in haploinsufficiency have been identified in a relatively large proportion of patients with common variable immunodeficiency (CVID) [10]. Therefore, the *NFKB1* intron variants that were detected in our case often cause CVID. CVID is a type of B-cell (antibody-deficiency) disorder in which defects in B cell proliferation and differentiation lead to immunoglobulin production disorder. CVID is a clinically and genetically heterogeneous disorder characterized by hypogammaglobulinemia, impaired production of specific antibodies, and susceptibility to various infections (e.g., recurrent infections of the respiratory and gastrointestinal tracts), categorized into non-X-linked immunodeficiency [11]. T lymphocyte subsets of CVID show decreased numbers of switched memory B cells and naive CD4+ T cells, which are characterized by markedly reduced serum concentrations of IgG, low levels of IgA and/or IgM, and poor or absent responses to immunization [12]. However, some patients with antibody-deficiency disorders have normal or only modestly reduced immunoglobulin levels [1]. CVID is often observed in adults. The patient in this case had PJP and CMV esophagitis, with T lymphocyte subsets showing decreased B lymphocytes and CD4+ T cells as well as hypoimmunoglobulinemia with the progression of the disease, which were consistent with the clinical manifestations of CVID. Moreover, as CMV esophagitis developed later during the course of disease, we observed progressive reductions in B lymphocyte and CD4+ T lymphocyte counts, in addition to reduced immunoglobulin levels and a general worsening of the patient’s condition. Therefore, we speculate that the lymphocyte and immunoglobulin levels were related to the patient’s serious infection and critical condition.

Gastrointestinal involvement has been reported in 20–60% of CVID cases, with infectious and non-infectious factors. Intermittent or persistent diarrhea is the most common gastrointestinal symptom [13]. Some patients may experience non-infectious diarrhea that does not respond to empiric antibiotic therapy. The specific mechanism has remained unclear, and cellular and humoral immune deficiencies leading to intestinal mucosal immune disorders cannot be excluded [14]. The clinical endoscopic and pathological manifestations of the gastrointestinal infection in CMV have been rarely reported. By describing our case, we hope to provide an enhanced understanding of this disease.

The complement is a multi-functional complex system of the innate immunity comprising more than 30 proteins which are produced mainly by the liver and consist of activators and inhibitors interacting with each other to form three pathways of activation (classical, alternative, and lectin) [15]. This system has an important role in host defense against infectious agents, in the removal of apoptotic cells and immune complexes, and in the modulation of the adaptive immune system. Bacterial infections and autoimmune diseases are clinical conditions that are most frequently associated with complement defects. C6 deficiency is the most common terminal complement component deficiency and is mainly seen in African American populations. C6 deficiency is also a high-risk factor for *Neisseria meningitidis* infection. The clinical manifestations include recurrent *N. meningitidis* infection with a high likelihood of recurrence [16,17], which may commonly appear in a specific age group (6 months to 2 years) that is vulnerable to *N. meningitidis* infection. Terminal complement deficiency may not appear until the second or third decade of life [17]. Patients may be screened for terminal complement deficiency by examining CH50. C6 deficiency is rarely reported in the Chinese population; the first case with a pathogenic gene mutation reported by Li et al. at the University of Hong Kong in 2020. The main clinical manifestation of the patient was recurrent meningococcal septicemia. Subsequent family screening and extensive clinical, serological, and genetic investigations were performed to identify the first Chinese patient with C6 deficiency (Guangzhou, China) [18]. There was no clinical evidence of *N. meningitidis* infection in the patient reported in our case, and the lack of early identification for PID resulted in an incomplete examination of CH50. The mother and younger brother of the patient were heterozygous carriers of nonsense mutations in C6, the same type of gene mutation as the patient. However, they had an unremarkable history of infections. Intronic *NFKB1* mutations were also observed in the patient’s father, who showed no clinical manifestations of repeated opportunistic infections. Furthermore, a search of the PubMed revealed no literature relating to the simultaneous mutation of C6 and *NFKB1* when screened with the following keywords: (C6 [Title/Abstract]) AND (NFKB1 [Title/Abstract]), (C6 [Title/Abstract]) AND (CVID [Title/Abstract]), (complement [Title/Abstract]) AND (NFKB1 [Title/Abstract]). We identified a few case reports, mainly relating to C2 deficiency associated with CVID for which a high degree of consanguinity was noted in the parents of the patient [19,20]. Therefore, simultaneous gene mutations of the nonsense C6 and intronic *NFKB1* were speculated to have contributed to the immunodeficiency in our case, with significant clinical implications. The clinical manifestations of the patient’s parents and younger brother will be followed up and further serological and genetic tests will be performed as necessary.

## 4 Conclusions

A diagnosis of PID should be suspected in patients with recurrent opportunistic infections, reduced CD4+ T and B lymphocyte counts, and hypoimmunoglobulinemia in the conditions that secondary immunodeficiency factors were excluded. Clinically, we should highlight the importance of a thorough history (current, past, personal, and family history) and physical examinations. In-depth investigations to clarify PID often involve genetic analysis, lymphocyte proliferation assays, flow cytometry, measurement of serum immunoglobulin (Ig) levels, assessment of serum specific antibody titers in response to vaccine antigens, neutrophil function assays, stimulation assays for cytokine responses, and complement studies [1]. It is important to not only clarify the presence of known familial mutations, but also to explore unknown pedigree analysis.
